# Developmental Relationships Between Early Vocabulary Acquisition, Joint Attention and Parental Supportive Behaviors

**DOI:** 10.1111/infa.70004

**Published:** 2025-02-07

**Authors:** Johan Wengman, Linda Forssman

**Affiliations:** ^1^ Department of Psychology Uppsala University Uppsala Sweden

**Keywords:** expressive vocabulary, joint attention, language acquisition, parent‐child interactions, receptive vocabulary

## Abstract

In late infancy and early toddlerhood, joint attention ability is widely recognized as a crucial foundation for children's vocabulary development, though the exact nature of its contribution remains debated. This study investigates associations between joint attention and subsequent vocabulary development, as well as the possible moderating role of supportive parental behaviors. Seventy children and their families participated in this longitudinal study, which began when the children were 10 months of age. Parents completed the Swedish Communicative Development Inventory (CDI) at four age points (10, 12, 18, and 24 months) to assess receptive and expressive vocabulary growth. Children participated in lab‐based assessment of joint attention abilities at 10, 12, and 18 months. Additionally, at 10 and 12 months, parent‐child dyads participated in two semi‐structured lab assessments to evaluate the quality of parental supportive behaviors during interactions with their child. Primary analysis showed no significant effects of joint attention on subsequent receptive and expressive vocabulary. However, a significant interaction was found between a child's ability to respond to joint attention cues and parental supportive behaviors on receptive vocabulary. These findings indicate that parental supportive behaviors play a crucial role in promoting the development of children's receptive vocabulary.

## 1 Introduction

Learning to understand and produce words is a major milestone in a child's development. This development is predominantly facilitated through social and communicative interactions. Within these social interactions, the child's ability to coordinate joint visual attention toward objects or events with a social partner is widely considered foundational for language development (e.g., Bruner 1975; Baldwin 1991, 1993; Carpenter et al. 1998; Morales et al. 2000). Several previous studies report correlations between children's proficiency in joint attention (JA) and their vocabulary size (e.g., Beuker et al. 2013; Brooks and Meltzoff 2008, 2015; Carpenter et al. 1998; Gliga et al. 2012; Morales et al. 2000).

Yet, a recent review challenges the empirical basis for these correlations (Astor and Gredebäck 2022), as the majority of studies reviewed on JA and language development report null findings. This underscores the need for further investigation into the role of JA in early vocabulary acquisition. In this paper, we propose that the variance observed in studies examining the relationship between JA and vocabulary development could stem from a combination of two factors: the specific aspect of JA measured, and the degree of parental skills in establishing effective language learning contexts.

Early word recognition typically begins between 6 and 9 months after birth (Bergelson and Swingley 2012; Tincoff and Jusczyk 1999), with first words often spoken around 12 months of age (Fenson et al. 2000). This period, the 6–12‐month span, is marked by a transition from dyadic (one‐on‐one) communication to synchronized triadic attention with others, leading to a shared focus of attention (Bruner 1975; Butterworth and Jarrett 1991; Brooks and Meltzoff 2008; Carpenter et al. 1998; Tomasello and Farrar 1986). These JA episodes can be initiated by either the child (e.g., through alternating gaze), so‐called initiating joint attention (IJA) or by their social partner (e.g., by following a point or eye gaze), where the child responds to bids for joint attention (RJA) (Dawson et al. 2004). It is theorized that the cultivation of JA skills significantly aids in linking spoken words to their meaning, thereby accelerating vocabulary acquisition (Brooks and Meltzoff 2015; Morales et al. 2000).

Initiating and responding to JA bids are understood to be distinct abilities, with separate contributions to early word learning (Adamson et al. 2009; Mundy et al. 2007). These abilities develop along separate timelines: the ability to RJA typically emerges around 6–12 months of age, while the ability to IJA usually develops slightly later, from 9 to 15 months (Carpenter et al. 1998).

RJA has been correlated to concurrent and subsequent development of receptive and expressive vocabulary during infancy and toddler years (Brooks and Meltzoff 2008; Morales et al. 2000; Mundy and Gomes 1998). RJA's role in language acquisition is believed to be particularly critical during the second year of life. During this period, episodes of shared attention, in which a knowledgeable adult verbally labels the focus of joint attention, provide a powerful mechanism for expanding the child's vocabulary (Masek et al. 2021). The child's ability to infer the object of the adult's attention through a combination of multimodal cues, such as gaze, pointing, and body orientation, is likely correlated to the frequency of such instances. However, its influence appears to peak and then stabilize around 18–24 months of age (Morales et al. 2000).

Conversely, the association between IJA and vocabulary development is more ambiguous. It is plausible that children who independently initiate JA episodes may encounter more opportunities to acquire new vocabulary (Akhtar 1991). Some studies suggest a more direct link between IJA and subsequent vocabulary growth (Mundy et al. 2007; Shih et al. 2021). However, these associations may be more pronounced for certain types of IJA, such as high‐level, socially oriented IJA (e.g., showing, pointing), as opposed to low‐level gaze‐focused IJA (i.e., coordinated gaze shifts) (Mundy and Gomes 1998; Pickard and Ingersoll 2015).

Despite the recognized importance of JA in language development, the exact nature of its contribution remains a subject of debate. A recent review (Astor and Gredebäck 2022) on the role of gaze following in language development highlighted this uncertainty, revealing a weaker‐than‐expected link between JA and language skills. These findings suggest that the role of JA in language development may be overstated, potentially due to the research practice of conducting multiple statistical tests within and across different studies, which can increase the likelihood of false positives or inflated associations being reported as significant. Another potential explanation for the variation seen in research exploring the connection between JA and vocabulary development may be attributed to varying levels of parental proficiency in facilitating language learning during JA episodes.

Parental supportive behaviors, such as responding in a contingent and sensitive manner to a child's behavior, have consistently been linked to positive developmental outcomes, including enhanced vocabulary acquisition (e.g., Hirsh‐Pasek and Burchinal 2006; Madigan et al. 2019; Tamis‐LeMonda, Kuchirko, and Song 2014). Specifically, parental sensitivity, defined by attentive and responsive engagement with a child's cues and interests, has been associated with both receptive and expressive vocabulary development (Leigh, Nievar, and Nathans 2011). For instance, research indicates that parental sensitivity at 9 months predicts the child's receptive vocabulary at 13 months (Tamis‐LeMonda et al. 2001). Moreover, engaging in parental contingent language significantly increases the likelihood that labeling of an object takes place when a target object captures a child's focus (Masek et al. 2021; Smith, Suanda, and Yu 2014; Yurovsky, Smith, and Yu 2013). This highlights the critical role of attuned parental responsiveness in supporting effective vocabulary learning by closely observing and engaging with the child's focus of attention. To our knowledge, no prior studies have examined the interaction between joint attention and parental language supporting behaviors in predicting vocabulary development during the second year of life.

### 1.1 The Present Study

The aim of the present study was to investigate associations between children's (*n* = 70) joint attention and vocabulary development between the ages of 10 and 24 months, as well as the possible moderating role of supportive parental behaviors.

To do so, we measured the children's vocabulary development at 10, 12, 18 and 24 months, using parental reports on a Swedish short version of the MacArthur‐Bates Communicative Development Inventory (SE‐CDI; Eriksson, Westerlund, and Berglund 2002). At 10, 12, and 18 months we administered a lab‐based assessment of RJA and IJA from the Early Social Communication Scale (ESCS; Mundy et al. 2003). As a measure of the quality of supportive parental behaviors, we used two semi‐structured observation assessments of parent‐child interactions during book sharing (Kucirkova, Dale, and Sylva 2016) and object exploration (Whipple, Bernier, and Mageau 2011) at 10 and 12 months. Based on theory and previous research, we will test the following hypotheses:

Hypothesis 1 *Joint attention, including both RJA and IJA, at 10, 12, and 18 months of age will be positively associated with later receptive and expressive vocabulary at 12, 18, and 24 months of age*.

Hypothesis 2 *The associations between joint attention and vocabulary will be moderated by language supportive parental behaviors. Specifically, children who have better joint attention skills and parents who demonstrate better supportive behaviors will exhibit greater vocabulary growth*.

Variables known to potentially relate to language development were chosen as covariates in the analysis. These variables were child gender, child language status (other language than Swedish spoken at home), child general cognitive ability, and parental education (e.g., Berglund, Eriksson, and Westerlund 2005; Bleses et al. 2016; Hoff 2003).

## 2 Methods

### 2.1 Study Design, Recruitment, and Participants

This study is part of the “*The Roadmap to Executive function and Language*” (REaL) research project that followed children from 10 to 24 months of age in Uppsala, Sweden. The project consisted of two studies: a longitudinal study (*N* = 70), started in September 2019 and ended in July 2021, and a randomized controlled study (*N* = 115), started in January 2020 and ended in September 2022, registration number ISRCTN22319305. This study is based on data from the longitudinal study, which has not been published previously.

Participants were recruited through: (i) reaching out to families who had expressed an interest in participating in research with their child at the Uppsala Child and Baby Lab; (ii) inviting participants who were already participating in a population‐based study on perinatal mental health in Uppsala, Sweden (Axfors et al. 2019); (iii) distributing information about the study at child health care centers and open preschools in Uppsala; and (iv) advertising on social media platforms.

Before enrollment, caregivers who expressed interest in participating in the study with their child received detailed information about the study and were screened based on inclusion and exclusion criteria. To be eligible, families had to meet the following inclusion criteria: (i) consent from the child's caregiver(s) to study participation; (ii) the participating child was 10 months old (+/− 4 weeks) at the initial assessment; (iii) one of the child's caregiver's (with whom the child lived) spoke Swedish and the family spoke at least some Swedish at home in the child's presence; (iv) the same caregiver (mother or father) could attend the first two lab visits with their participating child (due to the inclusion of parent‐child semi‐structured observations at these points). Families were ineligible if they met any of the following exclusion criteria: (i) the child was born prematurely (less than 37 weeks gestational age) and/or (ii) the child had an illness or disability that could hinder full participation in the study.

**FIGURE 1:**
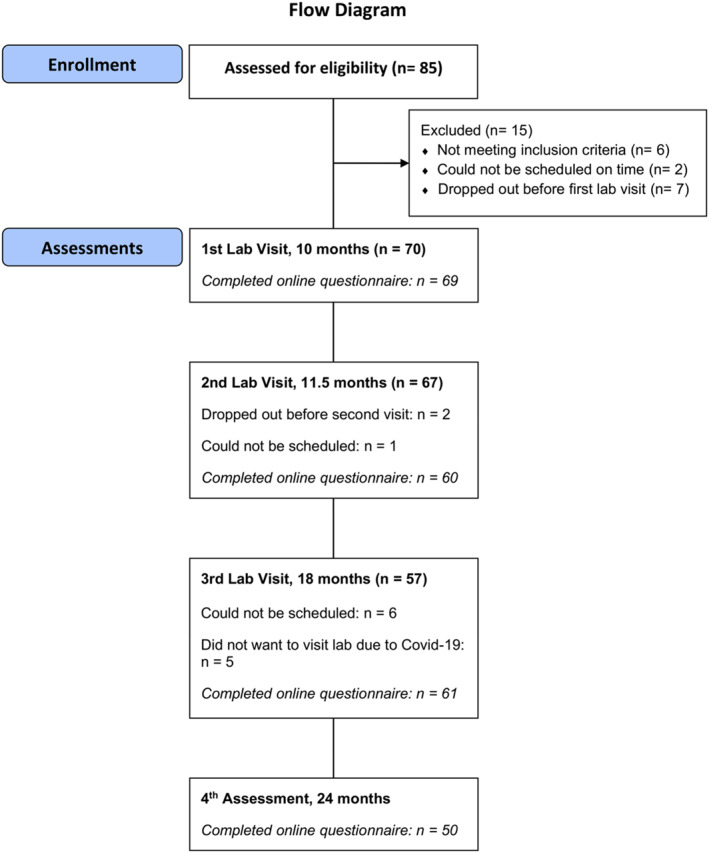
Diagram showing flow of participants through the study.

Data collection occurred at four time points, with the first three involving lab visits. At all four time points, participating caregivers completed a questionnaire online, which was created using Survey Monkey (https://sv.surveymonkey.com). The study's targeted sample size was 70 children and their families. The sample size was predetermined before enrollment and based on practical considerations, including the availability of participants within the targeted age range, the study's timeline, and available resources required for labor‐intensive lab assessments. Overall, 70 children (46% females) and their families participated in the study. The participants' first lab visit took place around 10 months of age (*M* = 309.41 days; *SD* = 17.28; range = 273–339), with a second visit scheduled about 6–7 weeks later (*M* = 48.11 days; *SD* = 8.42; range = 34–85), when they were approximately 11.5 months old (*M* = 357.03 days; *SD* = 19.56; range = 316–395). The third visit was planned for when the children reached 18 months (*M* = 545.40 days; *SD* = 17.91; range = 519–584). At 24 months of age (*M* = 737.48 days; *SD* = 12.21; range = 715–789), caregivers were invited to complete a questionnaire. While we aimed to adhere to this testing schedule, practical considerations, such as participant availability and rescheduling due to illness, occasionally resulted in slight deviations. See Table 1 for socio‐demographic characteristics of the sample. See Figure 1 for a participant flow diagram.

**TABLE 1:** Socio‐demographic description of sample.

	% (*n*)	*M* (*SD*)	Min‐max
Family structure^a^			
Child lives with both parents	97.10 (67)		
Child lives with mother	2.90 (2)		
Swedish language spoken at home^b^			
100% of the time	75.71 (53)		
75%–90% of the time	12.86 (9)		
20%–75% of the time	11.43 (8)		
Participating parent			
Mother	74.29 (52)		
Parental age in years^a^			
Participating parent		33.20 (4.86)	21–44
Other parent		34.55 (6.08)	22–55
Parental education^a^			
Participating parent			
High school diploma	17.39 (12)		
University/college	82.61 (57)		
Other parent			
Elementary school (9 years)	2.94 (2)		
High school diploma	19.12 (13)		
University/college	77.95 (53)		

^a^ Assessed through questionnaire in connection with first lab visit (response rate = 69/70).

^b^ Assesed during the screening process.

The present study was conducted according to guidelines laid down in the Declaration of Helsinki, with written informed consent obtained from a parent or guardian for each child before any assessment of data collection. All procedures involving human subjects in this study were approved by the Swedish Ethical Review Authority (Etikprövningsmyndigheten; reference number 2019–03140) Families received a gift voucher (∼10 €) per lab visit.

### 2.2 Measures and Procedures

The first two lab visits lasted approximately 75–90 min each (including breaks), while the third lab visit took about 60 min (including breaks). An online administered questionnaire was sent a week before each lab visit and at 24 months, which took about 60 min to complete. A full list of tasks used in the REaL project can be found at Databrary (Forssman 2022). Study outcome measures and covariates are described below and presented in Table 2.

**TABLE 2:** Study outcomes and covariate measures at 10 months (T1), 12 months (T2), 18 months (T3) and 24 months (T4), including number of valid assessments.

Outcomes	Measures	T1 (*n*)	T2 (*n*)	T3 (*n*)	T4 (*n*)
Expressive vocabulary	Parental report: CDI	(70)	(69)	(63)	(50)
Receptive vocabulary	Parental report: CDI	(70)	(69)	(63)	(50)
Responding to joint attention	Observation: ESCS	(62)	(65)	(50)	
Initiating joint attention	Observation: ESCS	(66)	(64)	(48)	
Parental support object exploration	Observation: PASS	(63)	(66)		
Parental support shared reading	Observation: BSSI	(67)	(61)		
Covariates					
Child cognition	Bayley III cognitive scale	(69)			
Child gender	Parental report	(70)			
Child language status	Parental report	(70)			
Parental education	Parental report	(70)			

*Note:* The number of children visiting the lab at T1, T2 and T3, were 70, 67, and 57, respectively.

Abbreviations: BSSI, Book Sharing Scale for Infants; CDI, The Swedish Early Communication Development Inventory; ESCS, Early Social Communication Scales; PASS, Parental Autonomy Support Scale.

#### 2.2.1 Child Language

Parental reports on the short version of the *Swedish CDI* (SE‐CDI; Eriksson, Westerlund, and Berglund 2002) were used at all four assessment points to measure *word production and word comprehension* (i.e., expressive and receptive vocabulary). We asked parents to mark (by placing a check next to each word) what their child currently understands or says in Swedish. We clarified that the pronunciation of the words did not matter, if the parents understood what their child meant. Aggregated scores for expressive and receptive vocabulary were calculated from this 90‐item checklist, with a range of 0–90. To include CDI data for a participant at a specific time point, a minimum response rate of at least 90% of the items was required.

#### 2.2.2 Child Joint Attention

Child joint attention was assessed using two tasks, the Object Spectacle task (IJA) and the Gaze Following task (RJA), drawn from the Early Social Communication Scales (ESCS; Mundy et al. 2003). For both tasks, the experimenter and the child sat at a table facing each other, with the child positioned in a high chair or on their parent's lap. The tasks were video recorded for later coding.

In the Object Spectacle task, the experimenter presented a toy to the child for 6 s, just out of their reach. This was repeated across 9 trials using different toys (a dog with a wagging tail, a moving hand puppet, and a shaking rattle). The child's number of alternating gazes between the toy and the experiment leader, during the 6‐s period, was calculated for every valid trial. A trial was deemed invalid if the child was out of position or did not pay attention to the experimenter when the trial commenced, if the experimenter made a sound before the child made eye contact, or if the child grabbed the toy before the end of the coding window. The outcome measure was the proportion of alternating gazes, calculated by averaging the number of alternating gazes across all valid trials.

Interrater reliability, based on a random subset of 20% of the data showed high agreement (ICC = 0.93). On average, the children provided 8.43 (*SD* = 0.98), 8.44 (*SD* = 1.24) and 7.50 (*SD* = 1.80) valid trials out of nine possible at the first, second and third lab assessment, respectively. To be included in the analyses, the child needed at least 6 valid trials. Several infants who participated in the lab assessments had missing data for this task due to experimenter or technical errors, or because they did not provide sufficient data for various reasons across the three time points (10 months: *n* = 4; 12 months: *n* = 3; 18 months: *n* = 9).

In the Gaze Following task, the experimenter sequentially pointed and gazed at four wall posters. Positioned to the left, right, behind and to the left, and behind and to the right of the child, each poster served as the target twice across 8 trials. In each trial, experimenter caught the child's attention, then pointed and gazed at poster for a total of 6 s. After 3 s the experimenter looked back at the child, said the child's name, and then turned and pointed to the poster for an additional 3 s. The trial ended with the experimenter naming the object on the poster (e.g., ‘that was a lion’). The child's number of correct gaze‐following responses (i.e., child looking in the correct direction beyond the index finger) was calculated for every valid trial. A trial was defined as invalid if the child was out of position or did not pay attention to the experiment leaders face or index finger when the trial commenced or the experimenter said “look” instead of the child's name. The outcome measure was the proportion of correct gaze following responses. Interrater reliability, based on a random subset of 20% of the data showed excellent agreement (ICC = 0.96). On average, the children provided 7.24 (*SD* = 2.28), 7.77 (*SD* = 1.03) and 6.90 (*SD* = 1.73) valid trials out of 8 at the first, second and third lab assessment, respectively. To be included in the analyses, at least 4 valid trials were needed. Several infants who participated in the lab assessments had missing data for this task due to experimenter or technical errors, or because they did not provide sufficient data for various reasons across the three time points (10 months: *n* = 8; 12 months: *n* = 2; 18 months: *n* = 7).

#### 2.2.3 Parental Supportive Behaviors

To assess the quality of parental supportive behaviors during parent‐child interactions, we used two semi‐structured observation tasks at the first and second lab visit. The tasks included parent‐child interactions during object exploration and book sharing. Based on the assumption that both observation tasks reflect relatively stable and time independent constructs (see e.g., Matte‐Gagné et al. 2013), the average of the scores from both lab visits will be used in the analysis. In the rare instances where the tested parent was not the same at both visits, the value from the second lab visit will be used.

**Object exploration.** The assessment and coding were based on the Parental Autonomy Support Scale (Whipple, Bernier, and Mageau 2011). During this assessment, the parent and child were seated together at a table. The dyad was presented with a challenging shape‐sorting toy and instructed to explore the toy together, then the experimenter left the room for 4 min. Parental supportive behaviors were coded from video recordings using four scales ranging from 1 (not supportive) to five (extremely supportive): (1) *Supports the Child's Autonomy—*by adapting the task to create an optimal challenge for the child; (2) *Verbalization Toward the Child—by encouraging the child, giving useful hints and using a tone of voice that indicates that the parent is there to help;* (3) *Flexibility in Keeping the Child on Task and Takes the Child's Perspective—by demonstrating flexibility in keeping the child on task and taking the child's perspective*, and (4) *Following the Child's Pace*—by letting the child set the pace, giving the child opportunities to make choices and play an active role in the task.

The mean scores from the first and second assessment points were *M* = 2.65 (*SD* = 0.62) and *M* = 3.01 (*SD* = 0.77), respectively. Interrater reliabilities, established by intra‐class correlation for randomly selected subsets of 20% interactions per lab assessment, were moderate to good (first assessment: ICC = 0.81; second assessment: ICC = 0.65). The scales were borderline significantly correlated (*r* = 0.25, *p* = 0.051) between the two assessment points and averaged into one score (Cronbach's *α* = 0.86).

**Book Sharing.** The assessment was coded using the Book Sharing Scale for Infants (BSSI; Kucirkova, Dale, and Sylva 2016). In this assessment, the parent and child sat on a soft mattress in the lab room. They were presented with a picture‐book (‘Miffy på bondgården’ by D. Bruna, 2011) and instructed to explore the book together. After the book was handed to the parent, the experimenter left the room for 4 min. Parental book sharing behaviors were coded from video recordings and based on 14 characteristics of behavior at three levels (1 = low, 2 = moderate, 3 = high). For example, the characteristics included demonstrating pleasure in reading to the child, responding to the child's communication cues, using repetition, pointing/prompts, praising efforts, and scaffolding (e.g., labeling, elaboration, decontextualization).

The mean scores from the first and second assessment points were *M* = 2.03 (*SD* = 0.30) and *M* = 2.04 (*SD* = 0.33), respectively. Interrater reliabilities, established by intra‐class correlation for randomly selected subsets of 20% interactions per assessment, were moderate to excellent (first assessment: ICC = 0.64; second assessment: ICC = 0.93). The scales were significantly correlated (*r* = 0.70, *p* < 0.001) between the two assessment points and were averaged into one score (Cronbach's *α* = 0.80).

#### 2.2.4 Covariates

Child cognition was assessed with the Bayley III Cognitive Scale (Bayley 2006) during the first lab visit. Child characteristics (e.g., child gender as perceived by caregivers) and socio‐demographic background information were collected during screening and through an online parental questionnaire preceding the first lab visit.

### 2.3 Data Analysis

All statistical analyses were performed in SPSS version 28. The variables selected and the data analysis plan were pre‐registered before any data analyses were conducted, see pre‐registration https://doi.org/10.17605/OSF.IO/4QJN9. Prior to our main analysis, all data were checked for normality, including skewness and kurtosis, visual inspections of Q‐Q plots, and multivariate tests of normality, to ensure that parametric statistics were valid. The two scales assessing parental supportive behaviors, PASS and BSSI, were examined for reliability, and following standardization, we combined the outcomes from the two scales into one average measure of parental supportive behavior. Traditional alpha levels of *p* < 0.05 (significant) were used.

Based on two initial null‐models including just dependent variables and *participant* as random factor, four possible covariates were assessed. Specifically, child gender, child language status, parental education and general cognitive development were included as fixed‐effect covariates in all subsequent models if they were significant predictor in either null‐model. All mixed models were conducted using restricted maximum likelihood estimation, type‐III sum of squares and Satterthwaite approximations. In all mixed models the dependent variable for all subsequent analyses used the cross‐lagged scores on the CDI that is, the score from the subsequent time‐point. To maintain model simplicity, only random intercepts were included, excluding random slopes. This approach aligns with the view that children's vocabulary development generally follows similar patterns between 10 and 24 months, despite variations in the age at which development begins. See Table SI1 for a description of the models tested.

## 3 Results

### 3.1 Preliminary Analysis

Before proceeding with our primary analysis, missing data was dealt with through imputation (estimation maximization). After concluding that Little's MCAR test was not significant (*Chi‐Square* = 257.47, *p* = 0.445), estimation maximization was used to impute missing data for all outcome variables seen in Table 2. Descriptive statistics for all study variables are displayed in Table 3.

**TABLE 3:** Mean, standard deviations for primary outcome variables (*n* = 70).

	10 months	12 months	18 months	24 months
	*M* (*SD*)	*M* (*SD*)	*M* (*SD*)	*M* (*SD*)
Rec	23.72 (15.98)	38.09 (19.78)	69.86 (12.88)	84.92 (5.85)
Exp	2.68 (3.93)	5.09 (5.70)	26.02 (21.09)	70.88 (18.79)
RJA	0.26 (0.18)	0.35 (0.24)	0.72 (0.27)	
IJA	0.50 (0.38)	0.60 (0.36)	0.65 (0.35)	
PSB^a^	0.00 (0.81)	0.00 (0.81)		
Bayley	9.97 (2.26)			

Abbreviations: Bayley, Bayley III cognitive subscale; Exp, CDI expressive vocabulary; IJA, Initiating Joint Attention; PSB, Parental Supportive Behavior; Rec, CDI receptive vocabulary; RJA, Responding to Joint Attention.

^a^ Parental Supportive Behavior is a standardized measure, averaged across the first two assessments (T1 and T2).

Zero‐order correlations for the primary variables are displayed in Table 4. Zero‐order correlations showed expected positive correlations between vocabulary measures across time points. All vocabulary measures, except receptive vocabulary at 12 months and expressive vocabulary at 24 months, were significantly correlated.

**TABLE 4:** Pearson's correlations between study outcome variables at 10, 12, 18 and 24 months of age.

	Receptive				Expressive				RJA			IJA			PSB
	1.	2.	3.	4.	5.	6.	7.	8.	9.	10.	11.	12.	13.	14.	15.
Age	*10*	*12*	*18*	*24*	*10*	*12*	*18*	*24*	*10*	*12*	*18*	*10*	*12*	*18*	*10*, *12*
1.	—														
2.	0.85**	—													
3.	0.53**	0.64**	—												
4.	0.55**	0.49**	0.85**	—											
5.	0.59**	0.60**	0.48**	0.40**	—										
6.	0.55**	0.58**	0.41**	0.37**	0.88**	—									
7.	0.56**	0.52**	0.66**	0.70**	0.64**	0.52**	—								
8.	0.25*	0.19	0.50**	0.69**	0.30*	0.30*	**0**.63**	—							
9.	0.19	0.18	0.23	0.20	0.10	0.17	**0**.24*	0.16	—						
10.	0.31*	**0**.32**	0.29*	0.19	0.31**	0.44**	0.08	−0.01	0.54**	—					
11.	−0.01	−0.04	−0.01	−0.02	−0.08	0.19	−0.03	0.00	0.02	0.33**	—				
12.	−0.03	0.07	−0.04	−0.02	−0.02	0.06	−0.08	−0.07	−0.08	0.08	0.20	—			
13.	0.13	0.08	0.13	0.12	0.16	0.14	0.13	0.16	−0.03	0.11	0.19	0.39**	—		
14.	0.19	0.18	0.06	0.23	0.20	0.29*	0.15	0.17	−0.08	0.08	0.20	0.43**	0.28*	—	
15.	0.13	0.15	0.21	0.31*	0.25*	0.18	0.16	0.07	0.15	0.13	−0.05	−0.18	0.01	0.27*	

Abbreviations: Expressive, CDI expressive vocabulary; IJA, Initiating joint attention; PSB, Parental Supportive Behavior; Receptive, CDI receptive vocabulary; RJA, Responding to joint attention.

*Correlation is significant at the 0.05 level (2‐tailed). **Correlation is significant at the 0.01 level (2‐tailed).

There were several significant correlations between CDI and JA measures. More specifically, RJA correlated positively with receptive and expressive language at 10 and 12 months, and with receptive vocabulary at 18 months. There were few significant correlations between vocabulary measures and IJA and parental supportive behaviors.

Notably, assessments of RJA and IJA showed positive and significant correlations across time points, within each construct, whereas the two JA constructs were not significantly correlated with each other at any time point.

#### 3.1.1 Test of Control Variables

Two initial models with cross‐lagged vocabulary measures (receptive or expressive vocabulary), random intercepts for participants and the covariates were constructed. As none of the covariates significantly predicted vocabulary measures (*p*s > 0.29), they were not included in subsequent models.

### 3.2 Primary Analysis

#### 3.2.1 Joint Attention and Vocabulary Development

Four mixed models were constructed, one for each pairing of the dependent variables (i.e., receptive vocabulary and expressive vocabulary), and the main independent variables of interest (i.e., RJA and IJA), to test the first hypothesis that JA at 10, 12 and 18 months of age would be positively associated with subsequent receptive and expressive vocabulary, that is, at 12, 18 and 24 months of age. All models included random intercepts for participants, age (as measured by lab visit 1, 2 and 3) as a fixed effect and an interaction between age and the JA measures (i.e., RJA and IJA). Summarized results for all four models are shown in Table 5.

**TABLE 5:** Regression coefficients for mixed models. Model 1 to 4 display effects of responding to joint attention (RJA) and initiating joint attention (IJA) on receptive and expressive vocabulary.

Predictor	Receptive vocabulary			Expressive vocabulary		
	Model 1			Model 2		
	*df*	*F*	Sig.	*df*	*F*	Sig.
Intercept	1, 177.62	823.14	< 0.001	1, 178.35	169.59	< 0.001
Age	2, 152.52	82.14	< 0.001	2, 154.34	63.39	< 0.001
RJA	1, 196.87	3.84	0.052	1, 199.88	0.05	0.822
Age × RJA	2, 158.21	1.04	0.358	2, 160.45	0.41	0.665

Predictor	Model 3			Model 4		
	*df*	*F*	Sig.	*df*	*F*	Sig.
Intercept	1, 164.38	959.18	< 0.001	1, 157.06	165.00	< 0.001
Age	2, 160.13	109.56	< 0.001	2, 152.34	110.19	< 0.001
IJA	1, 204.00	1.28	0.260	1, 203.42	3.00	0.085
Age × IJA	2, 164.56	0.26	0.775	2, 157.53	0.04	0.938

Abbreviations: IJA, Initiating Joint Attention; PSB, Parental supportive behaviors; RJA, Responding to Joint Attention.

In all models, age was a significant predictor, with higher age predicting larger expressive and receptive vocabulary, independently of JA. However, we found no significant main effect of RJA or IJA on vocabulary measures (Models 1–4). There were also no significant interactions between JA measures and age (Models 1–4).

#### 3.2.2 Joint Attention, Vocabulary Development and the Moderating Role of Parental Supportive Behaviors

To test the hypothesis that associations between joint attention and vocabulary are moderated by supportive parental behaviors, four additional models were tested. These models were identical to the first four but with the inclusions of the variable parental supportive behaviors as a fixed effect and an interaction between parental supportive behaviors and the JA measures (i.e., RJA and IJA). Summarized results for all four models, Model 1 to Model 4, are shown in Table 6.

**TABLE 6:** Regression coefficients for mixed models. Model 1 to 4 display main effects and moderating effects of parental supportive behaviors on the associations between joint attention and vocabulary measures.

Predictor	Receptive vocabulary			Expressive vocabulary		
	Model 1			Model 2		
	*df*	*F*	Sig.	*df*	*F*	Sig.
Intercept	1, 185.85	854.69	< 0.001	1, 174.78	171.76	< 0.001
Age	2, 160.08	218.52	< 0.001	2, 152.62	230.48	< 0.001
RJA	1, 204	2.15	0.14	1, 199.25	0.01	0.943
PSB	1, 182.76	8.04	0.005	1, 177.12	0.37	0.543
RJA × PSB	1, 170.79	4.69	0.032	1, 165.07	0.09	0.771

Predictor	Model 3			Model 4		
	*df*	*F*	Sig.	*df*	*F*	Sig.
Intercept	1, 165.95	984.62	< 0.001	1, 155.59	167.31	< 0.001
Age	2, 147.09	366.56	< 0.001	2, 140.23	442.27	< 0.001
IJA	1, 204	1.83	0.178	1, 203.34	2.89	0.091
PSB	1, 169.52	6.64	0.011	1, 161.32	0.74	0.392
IJA × PSB	1, 201.12	3.20	0.075	1, 196.31	0.008	0.928

Abbreviations: IJA, Initiating Joint Attention; PSB, Parental supportive behaviors; RJA, Responding to Joint Attention.

**FIGURE 2:**
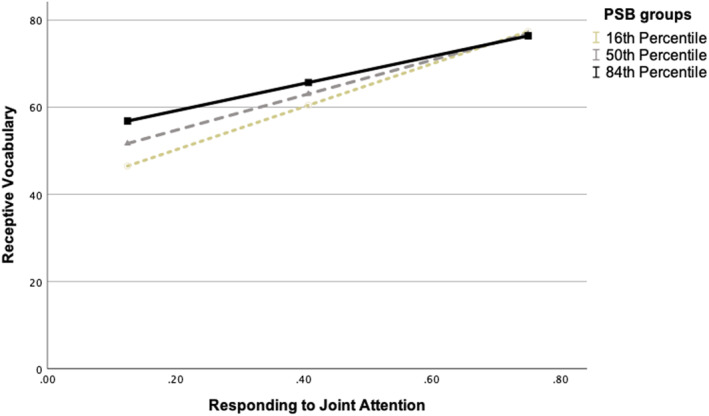
The conditional effect of responding to joint attention (RJA) on receptive vocabulary as a function of parental supportive behaviors (PSB). Receptive Vocabulary represent number of words the child understands; responding to joint attention represent proportion of correct responses in the gaze following task. The slopes represent the 16th, 50th, and 84th percentile of Parental Supportive Behaviors (PSB).

There were significant positive main effects of parental supportive behaviors on receptive vocabulary (see Models 1 and 3) and a significant interaction between RJA and parental supportive behaviors on receptive vocabulary (Model 1). The significant interaction effect is illustrated in Figure 2 and Table SI2 presents the conditional effects of RJA on receptive vocabulary across varying levels of PSB. Using the Johnson‐Neyman method, no significant transition points within the observed range of the moderator was found. No significant main effects or interactions were observed for IJA, nor were any significant effects found when expressive vocabulary was used as the dependent variable.

## 4 Discussion

The aim of this study was to examine the associations between children's JA abilities and vocabulary development between the ages of 10 and 24 months, as well as to investigate the potential moderating role of supportive parental behaviors. While previous studies have reported correlations between young children's JA proficiency and vocabulary size (e.g., Beuker et al. 2013; Brooks and Meltzoff 2008, 2015; Carpenter et al. 1998; Gliga et al. 2012; Morales et al. 2000), recent critiques (Astor and Gredebäck 2022) have called these findings into question, necessitating further examination. Our study found limited evidence supporting the role of individual differences in JA in relation to vocabulary development within this age range. However, the quality of parental supportive behavior during parent‐child interactions emerged as a significant factor. These findings contribute valuable insights to the understanding of early vocabulary development and suggest that strategies to enhance language acquisition in young children should focus on improving the quality of parent‐child interactions rather than emphasizing individual differences in JA.

### 4.1 Associations Between Vocabulary and Joint Attention Development

The first objective of this study was to determine whether JA, including both RJA and IJA, would be positively associated with subsequent receptive and expressive vocabulary. Our pre‐registered hypothesis anticipated that JA at 10, 12, and 18 months would be positively associated with later vocabulary outcomes. However, the findings provided no support for this hypothesis. Although preliminary correlational analysis revealed several significant associations, particularly between RJA and receptive vocabulary, these associations were not robust in our primary analysis, which controlled for age. Age consistently emerged as a significant predictor across all models, with children demonstrating larger vocabularies at later assessment points, independently of JA. This aligns with the expected progression of vocabulary growth as children mature.

The development of JA skills has been suggested as playing a crucial role in helping children link spoken words to their meaning, thereby facilitating vocabulary acquisition (Brooks and Meltzoff 2015; Morales et al. 2000). While it is plausible that children's growing social abilities, including JA, support language learning from their social environment, our findings indicate that individual differences in JA, beyond the effects of age, have limited explanatory power for vocabulary development.

An interesting observation regarding RJA and IJA was that these measures showed positive and significant correlations across all three assessment points, yet they did not correlate with each other at any single time point. This suggests that while both RJA and IJA are stable over time, they may represent distinct aspects of joint attention, consistent with previous research (e.g., Moore and Corkum 1994). It is possible that RJA primarily involves attention orienting skills, which are closely related to the ability to shift attention based on external cues, such as another person's gaze or point. This aligns with research suggesting that RJA taps into basic attentional mechanisms (e.g., Forssman and Wass 2018). In contrast, IJA may more strongly reflect aspects of social motivation, as it involves the child actively seeking to engage another person's attention for the purpose of sharing an experience, for example, through eye‐contact (e.g., Mundy 2018).

Although this study found no support for the association between JA and vocabulary outcomes, it's plausible that variations in parental proficiency in facilitating language learning during social interactions—particularly those involving JA episodes—could explain inconsistencies observed in research exploring the JA‐vocabulary connection.

### 4.2 The Role of Parental Supportive Behaviors

The second objective of this study was to investigate the potential moderating role of supportive parental behaviors. Consistent with our hypothesis, we found a significant interaction between parental supportive behaviors, as measured during parent‐child interaction tasks, and JA in relation to receptive vocabulary outcomes. However, this significant interaction was only found for RJA and not IJA. Additionally, there was a positive main effect of parental supportive behavior on receptive vocabulary. These findings resonate with previous studies showing that parental supportive behaviors, characterized by contingency, sensitivity, engagement and scaffolding, are linked to receptive vocabulary development (Leigh Nievar, and Nathans 2011; Tamis‐LeMonda et al. 2001). One possible explanation for this is that responsive parents may work harder to help the child identify the target of their attention. Although some parental behaviors may interact with a child's ability to initiate joint attention (IJA) in shaping vocabulary development, our findings suggest that the specific supportive behaviors examined in this study do not have a significant interaction with IJA.

In the context of vocabulary learning, parents who are adept at attuning to their child's focus of attention may more frequently label objects that capture the child's interest (Masek et al. 2021; Smith, Suanda, and Yu 2014; Yurovsky, Smith, and Yu 2013). We think it is likely that parental sensitivity to a child's focus of attention extends beyond nouns to also support verb acquisition. Parents who are more attuned to their child's focus of attention may provide rich verbal descriptions of ongoing actions that the child is observing or performing, thereby helping their child map words to actions (i.e., verbs). Based on our findings, we propose that while a child's growing ability to share attention creates learning opportunities, the caregiver's capacity to capitalize on these naturally occurring interactions is more critical for vocabulary acquisition than the child's individual JA skills. This highlights the importance of early interactions aimed at enhancing parents' language‐supportive behaviors to promote children's vocabulary growth. Practical recommendations to parents, such as book‐sharing guidelines, often advise using gestures or physical demonstrations to reinforce action words, thereby facilitating word learning.

While our results provide evidence that parental supportive behaviors are important for the development of receptive vocabulary, they do not explain why we did not observe similar outcomes for expressive vocabulary. One possible explanation is that expressive vocabulary relies on additional mechanisms beyond those required for receptive language, such as articulatory control, memory, and recall (Marchman and Fernald 2008). These mechanisms may be less directly influenced by parental behaviors. For instance, research by McGillion et al. (2017) demonstrated that the onset of babbling in infants serve as a strong predictor for the onset of first words, suggesting that infants' vocal practice facilitates the development of word production. Another possibility is that the later onset of development of expressive vocabulary (as is evident in Table 3) means these early parental behaviors are less influential. Future studies could benefit from follow‐up assessments to explore whether these null findings hold as children's language abilities mature.

### 4.3 Limitations

The study's findings should be interpreted in the context of the methodological choices that were made. One possible methodological limitation of our study is the reliance on parental reports, specifically the CDI, to assess child vocabulary development. Although previous research has demonstrated good test‐retest reliability and external validity for the CDI in children aged 8–30 months (e.g., Eriksson, Westerlund, and Berglund 2002; Fenson et al. 2000; Bornstein and Putnick 2012; DeMayo et al. 2021), it remains an indirect measure of the child's vocabulary. Parental reports may be subject to bias, as parents might either overestimate or underestimate certain aspects of their child's language abilities, particularly in assessing receptive vocabulary. Direct observations of children's word comprehension at this age typically rely on gaze or manual behavior, and these methods come with their own methodological challenges. Similarly, recording children's spontaneous speech, particularly in a lab setting, also poses limitations. As parental reports are based on daily interactions with their child, they may provide an advantage over these methods at early ages.

Another potential methodological limitation is our use of relatively brief lab‐based tasks to assess child joint attention and parental supportive behaviors. While previous research show that these tasks predict later developmental outcomes (e.g., Marciszko et al. 2020; Mundy et al. 2007), structured lab‐based tasks may not fully reflect interactions that occur in naturalistic, real‐world settings.

Additionally, the characteristics of our sample limit the generalizability of our findings. Despite our efforts to recruit a diverse population, most children came from homes with university‐educated parents, which may not represent the broader population. This is a demographic known to be associated with higher levels of parental engagement and enriched home learning environments, which have been associated with stronger language outcomes in previous research (e.g., Hoff 2003). As a result, our findings may not fully capture the variability in language development present in populations with more diverse socioeconomic backgrounds.

This sample characteristic may also partly explain why the study covariates—gender, language status, cognitive ability, and parental education—were not significantly associated with child language outcomes in our analysis. In a sample of children from families with relatively high educational backgrounds, these variables might have lesser of an observable impact.

## 5 Conclusion

In this study, we explored the associations between joint attention (JA) and vocabulary development during infancy and toddlerhood, as well as the potential moderating effects of supportive parental behaviors. Our primary analyses revealed no significant direct effects of JA on subsequent receptive and expressive vocabulary at 12, 18, and 24 months of age. However, we identified a significant interaction between a child's responsiveness to joint attention cues and parental supportive behaviors on receptive vocabulary outcomes.

These findings suggest that supportive parental behaviors play a crucial role in enhancing a child's receptive vocabulary. This underscores the importance of considering the broader social context, including parental interactions, in understanding early language development. Future research should further investigate these dynamics to better inform parental interventions aimed at promoting optimal language outcomes in young children.

## Author Contributions

**Johan Wengman:** data curation, formal analysis, investigation, methodology, writing–original draft, writing–review and editing. **Linda Forssman:** conceptualization, funding acquisition, methodology, project administration, resources, supervision, writing–original draft, writing–review and editing.

## Conflicts of Interest

The authors declare no conflicts of interest.

## Supporting information

Supporting Information S1

## Data Availability

Data request may be sent to the PI of this project at linda.forssman@psyk.uu.se.

## References

[infa70004-bib-0001] Adamson, L. B. , R. Bakeman , D. F. Deckner , and M. Romski . 2009. “Joint Engagement and the Emergence of Language in Children With Autism and Down Syndrome.” Journal of Autism and Developmental Disorders 39, no. 1: 84–96. 10.1007/s10803-008-0601-7. 18581223 PMC2640949

[infa70004-bib-0002] Akhtar, N. , F. Dunham , and P. J. Dunham . 1991. “Directive Interactions and Early Vocabulary Development: The Role of Joint Attentional Focus.” Journal of Child Language 18, no. 1: 41–49. 10.1017/s0305000900013283. 2010504

[infa70004-bib-0003] Astor, K. , and G. Gredebäck . 2022. “Gaze Following in Infancy: Five Big Questions That the Field Should Answer.” Advances in Child Development and Behavior 63: 191–223. 10.1016/bs.acdb.2022.04.003. 35871822

[infa70004-bib-0004] Axfors, C. , E. Bränn , H. E. Henriksson , et al. 2019. “Cohort Profile: The Biology, Affect, Stress, Imaging and Cognition (BASIC) Study on Perinatal Depression in a Population‐Based Swedish Cohort.” BMJ Open 9, no. 10: e031514. 10.1136/bmjopen-2019-031514. PMC6830667 31641004

[infa70004-bib-0005] Baldwin, D. A. 1991. “Infants' Contribution to the Achievement of Joint Reference.” Child Development 62, no. 5: 875–890. 10.1111/j.1467-8624.1991.tb01577.x. 1756664

[infa70004-bib-0006] Baldwin, D. A. 1993. “Infants' Ability to Consult the Speaker for Clues to Word Reference.” Journal of Child Language 20, no. 2: 395–418. 10.1017/s0305000900008345. 8376476

[infa70004-bib-0007] Bayley, N. 2006. Bayley Scales of Infant and Toddler Development (3rd ed.). San Antonio, TX: Pearson.

[infa70004-bib-0008] Bergelson, E. , and D. Swingley . 2012. “At 6–9 Months, Human Infants Know the Meanings of Many Common Nouns.” Proceedings of the National Academy of Sciences 109, no. 9: 3253–3258. 10.1073/pnas.1113380109. PMC3295309 22331874

[infa70004-bib-0009] Berglund, E. V. A. , M. Eriksson , and M. Westerlund . 2005. “Communicative Skills in Relation to Gender, Birth Order, Childcare and Socioeconomic Status in 18‐Month‐Old Children.” Scandinavian Journal of Psychology 46, no. 6: 485–491. 10.1111/j.1467-9450.2005.00480.x. 16277649

[infa70004-bib-0010] Beuker, K. T. , N. N. Rommelse , R. Donders , and J. K. Buitelaar . 2013. “Development of Early Communication Skills in the First Two Years of Life.” Infant Behavior and Development 36, no. 1: 71–83. 10.1016/j.infbeh.2012.11.001. 23261791

[infa70004-bib-0011] Bleses, D. , G. Makransky , P. S. Dale , A. Højen , and B. A. Ari . 2016. “Early Productive Vocabulary Predicts Academic Achievement 10 Years Later.” Applied PsychoLinguistics 37, no. 6: 1461–1476. 10.1017/s0142716416000060.

[infa70004-bib-0012] Bornstein, M. H. , and D. L. Putnick . 2012. “Stability of Language in Childhood: A Multiage, Multidomain, Multimeasure, and Multisource Study.” Developmental Psychology 48, no. 2: 477–491. 10.1037/a0025889. 22004343 PMC3412562

[infa70004-bib-0013] Brooks, R. , and A. N. Meltzoff . 2008. “Infant Gaze Following and Pointing Predict Accelerated Vocabulary Growth Through Two Years of Age: A Longitudinal, Growth Curve Modeling Study.” Journal of Child Language 35, no. 1: 207–220. 10.1017/s030500090700829x. 18300435

[infa70004-bib-0014] Brooks, R. , and A. N. Meltzoff . 2015. “Connecting the Dots From Infancy to Childhood: A Longitudinal Study Connecting Gaze Following, Language, and Explicit Theory of Mind.” Journal of Experimental Child Psychology 130: 67–78. 10.1016/j.jecp.2014.09.010. 25462032 PMC7089676

[infa70004-bib-0015] Bruner, J. S. 1975. “The Ontogenesis of Speech Acts.” Journal of Child Language 2, no. 1: 1–19. 10.1017/s0305000900000866.

[infa70004-bib-0016] Butterworth, G. , and N. Jarrett . 1991. “What Minds Have in Common Is Space: Spatial Mechanisms Serving Joint Visual Attention in Infancy.” British Journal of Developmental Psychology 9, no. 1: 55–72. 10.1111/j.2044-835x.1991.tb00862.x.

[infa70004-bib-0017] Carpenter, M. , K. Nagell , M. Tomasello , G. Butterworth , and C. Moore . 1998. “Social Cognition, Joint Attention, and Communicative Competence From 9 to 15 Months of Age.” Monographs of the Society for Research in Child Development 63, no. 4: i‐174. 10.2307/1166214. 9835078

[infa70004-bib-0018] Dawson, G. , K. Toth , R. Abbott , et al. 2004. “Early Social Attention Impairments in Autism: Social Orienting, Joint Attention, and Attention to Distress.” Developmental Psychology 40, no. 2: 271–283. 10.1037/0012-1649.40.2.271. 14979766

[infa70004-bib-0019] DeMayo, B. , D. Kellier , M. Braginsky , et al. 2021. “Web‐CDI: A System for Online Administration of the MacArthur‐Bates Communicative Development Inventories.” Language Development Research.

[infa70004-bib-0020] Eriksson, M. , M. Westerlund , and E. Berglund . 2002. “A Screening Version of the Swedish Communicative Development Inventories Designed for Use With 18‐Month‐Old Children.” Journal of Speech, Language, and Hearing Research 45, no. 5: 948–960. 10.1044/1092-4388(2002/077). 12381052

[infa70004-bib-0021] Fenson, L. , S. Pethick , C. Renda , J. L. Cox , P. S. Dale , and J. S. Reznick . 2000. “Short‐Form Versions of the MacArthur Communicative Development Inventories.” Applied PsychoLinguistics 21, no. 1: 95–116. 10.1017/s0142716400001053.

[infa70004-bib-0022] Forssman, L. 2022. REaL. Databrary: Retrieved April 24, 2023 from. https://nyu.databrary.org/volume/1506.

[infa70004-bib-0023] Forssman, L. , and S. V. Wass . 2018. “Training Basic Visual Attention Leads to Changes in Responsiveness to Social‐Communicative Cues in 9‐month‐olds.” Child Development 89, no. 3: e199–e213. 10.1111/cdev.12812. 28436545

[infa70004-bib-0024] Gliga, T. , M. Elsabbagh , K. Hudry , T. Charman , M. H. Johnson , and BASIS team . 2012. “Gaze Following, Gaze Reading, and Word Learning in Children at Risk for Autism.” Child Development 83, no. 3: 926–938. 10.1111/j.1467-8624.2012.01750.x. 22462503

[infa70004-bib-0025] Hirsh‐Pasek, K. , and M. Burchinal . 2006. “Mother and Caregiver Sensitivity Over Time: Predicting Language and Academic Outcomes With Variable‐And Person‐Centered Approaches.” Merrill‐Palmer Quarterly 52, no. 3: 449–485. 10.1353/mpq.2006.0027.

[infa70004-bib-0026] Hoff, E. 2003. “The Specificity of Environmental Influence: Socioeconomic Status Affects Early Vocabulary Development Via Maternal Speech.” Child Development 74, no. 5: 1368–1378. 10.1111/1467-8624.00612. 14552403

[infa70004-bib-0027] Kucirkova, N. , P. S. Dale , and K. Sylva . 2016. “Parents Reading With Their 10‐Month‐Old Babies: Key Predictors for High‐Quality Reading Styles.” Early Child Development and Care 0, no. 0: 1–13. 10.1080/03004430.2016.1211117.

[infa70004-bib-0028] Leigh, P. , M. A. Nievar , and L. Nathans . 2011. “Maternal Sensitivity and Language in Early Childhood: A Test of the Transactional Model.” Perceptual and Motor Skills 113, no. 1: 281–299. 10.2466/10.17.21.28.pms.113.4.281-299. 21987927

[infa70004-bib-0029] Madigan, S. , H. Prime , S. A. Graham , et al. 2019. “Parenting Behavior and Child Language: A Meta‐Analysis.” Pediatrics 144, no. 4. 10.1542/peds.2018-3556. 31551396

[infa70004-bib-0030] Marchman, V. A. , and A. Fernald . 2008. “Speed of Word Recognition and Vocabulary Knowledge in Infancy Predict Cognitive and Language Outcomes in Later Childhood.” Developmental Science 11, no. 3: F9–F16. 10.1111/j.1467-7687.2007.00671.x. 18466367 PMC2905590

[infa70004-bib-0031] Marciszko, C. , L. Forssman , B. Kenward , M. Lindskog , M. Fransson , and G. Gredebäck . 2020. “The Social Foundation of Executive Function.” Developmental Science 23, no. 3: e12924. 10.1111/desc.12924. 31733012

[infa70004-bib-0032] Masek, L. R. , B. T. M. McMillan , S. J. Paterson , C. S. Tamis‐LeMonda , R. M. Golinkoff , and K. Hirsh‐Pasek . 2021. “Where Language Meets Attention: How Contingent Interactions Promote Learning.” Developmental Review: Developmental Review 60: 100961. 10.1016/j.dr.2021.100961.

[infa70004-bib-0033] Matte‐Gagné, C. , A. Bernier , and C. Gagné . 2013. “Stability of Maternal Autonomy Support Between Infancy and Preschool Age.” Social Development 22, no. 3: 427–443. 10.1111/j.1467-9507.2012.00667.x.

[infa70004-bib-0034] McGillion, M. , J. S. Herbert , J. Pine , et al. 2017. “What Paves the Way to Conventional Language? the Predictive Value of Babble, Pointing, and Socioeconomic Status.” Child Development 88, no. 1: 156–166. 10.1111/cdev.12671. 27859008

[infa70004-bib-0035] Moore, C. , and V. Corkum . 1994. “Social Understanding at the End of the First Year of Life.” Developmental Review 14, no. 4: 349–372. 10.1006/drev.1994.1014.

[infa70004-bib-0036] Morales, M. , P. Mundy , C. E. F. Delgado , et al. 2000. “Responding to Joint Attention Across the 6‐ Through 24‐Month Age Period and Early Language Acquisition.” Journal of Applied Developmental Psychology 21, no. 3: 283–298. 10.1016/s0193-3973(99)00040-4.

[infa70004-bib-0037] Mundy, P. 2018. “A Review of Joint Attention and Social‐Cognitive Brain Systems in Typical Development and Autism Spectrum Disorder.” European Journal of Neuroscience 47, no. 6: 497–514. 10.1111/ejn.13720. 28922520

[infa70004-bib-0038] Mundy, P. , J. Block , C. Delgado , Y. Pomares , A. V. Van Hecke , and M. V. Parlade . 2007. “Individual Differences and the Development of Joint Attention in Infancy.” Child Development 78, no. 3: 938–954. 10.1111/j.1467-8624.2007.01042.x. 17517014 PMC2654237

[infa70004-bib-0039] Mundy, P. , C. Delgado , J. Block , M. Venezia , A. Hogan , and J. Seibert . 2003. Early Social Communication Scales (ESCS). Coral Gables, FL: University of Miami.

[infa70004-bib-0040] Mundy, P. , and A. Gomes . 1998. “Individual Differences in Joint Attention Skill Development in the Second Year.” Infant Behavior and Development 21, no. 3: 469–482. 10.1016/s0163-6383(98)90020-0.

[infa70004-bib-0041] Pickard, K. E. , and B. R. Ingersoll . 2015. “Brief Report: High and Low Level Initiations of Joint Attention, and Response to Joint Attention: Differential Relationships With Language and Imitation.” Journal of Autism and Developmental Disorders 45, no. 1: 262–268. 10.1007/s10803-014-2193-8. 25035090

[infa70004-bib-0042] Shih, W. , S. Shire , Y.‐C. Chang , and C. Kasari . 2021. “Joint Engagement Is a Potential Mechanism Leading to Increased Initiations of Joint Attention and Downstream Effects on Language: JASPER Early Intervention for Children With ASD.” Journal of Child Psychology and Psychiatry and Allied Disciplines 62, no. 10: 1228–1235. 10.1111/jcpp.13405. 33768537 PMC9879144

[infa70004-bib-0043] Smith, L. B. , S. H. Suanda , and C. Yu . 2014. “The Unrealized Promise of Infant Statistical Word–Referent Learning.” Trends in Cognitive Sciences 18, no. 5: 251–258. 10.1016/j.tics.2014.02.007. 24637154 PMC4009695

[infa70004-bib-0044] Tamis‐LeMonda, C. S. , M. H. Bornstein , and L. Baumwell . 2001. “Maternal Responsiveness and Children's Achievement of Language Milestones.” Child Development 72, no. 3: 748–767. 10.1111/1467-8624.00313. 11405580

[infa70004-bib-0045] Tamis‐LeMonda, C. S. , Y. Kuchirko , and L. Song . 2014. “Why Is Infant Language Learning Facilitated by Parental Responsiveness?” Current Directions in Psychological Science 23, no. 2: 121–126. 10.1177/0963721414522813.

[infa70004-bib-0046] Tincoff, R. , and P. W. Jusczyk . 1999. “Some Beginnings of Word Comprehension in 6‐Month‐Olds.” Psychological Science 10, no. 2: 172–175. 10.1111/1467-9280.00127.

[infa70004-bib-0047] Tomasello, M. , and M. J. Farrar . 1986. “Joint Attention and Early Language.” Child Development 57, no. 6: 1454–1463. 10.2307/1130423. 3802971

[infa70004-bib-0048] Whipple, N. , A. Bernier , and G. A. Mageau . 2011. “Broadening the Study of Infant Security of Attachment: Maternal Autonomy‐Support in the Context of Infant Exploration.” Social Development 20, no. 1: 17–32. 10.1111/j.1467-9507.2010.00574.x.

[infa70004-bib-0049] Yurovsky, D. , L. B. Smith , and C. Yu . 2013. “Statistical Word Learning at Scale: The Baby's View Is Better.” Developmental Science 16, no. 6: 959–966. 10.1111/desc.12036. 24118720 PMC4443688

