# Supplementary material for: Developmental Relationships Between Early Vocabulary Acquisition, Joint Attention and Parental Supportive Behaviors
Source: Infancy. 2025 Feb 7;30(1):e70004. doi: 10.1111/infa.70004 (PMC11803541; doi:10.1111/infa.70004)
Supplement: Supplementary file 1 — Supporting Information S1 [file INFA-30-0-s001.docx]

**Supplementary files**

**Table SI1.**

*Mixed models tested*

| # | Model formulas |
| --- | --- |
| 1 | Rec.lang ~ RJA * age + (1\|subject) |
| 2 | Exp.lang ~ RJA * age + (1\|subject) |
| 3 | Rec.lang ~ IJA * age + (1\|subject) |
| 4 | Exp.lang ~ IJA * age + (1\|subject) |
| 5 | Rec.lang ~ RJA * PSB + RJA * age + (1\|subject) |
| 6 | Exp.lang ~ RJA * PSB + RJA * age + (1\|subject) |
| 7 | Rec.lang ~ IJA * PSB + IJA * age + (1\|subject) |
| 8 | Exp.lang ~ IJA * PSB + IJA * age + (1\|subject) |

*Note.* Rec.lang = Receptive vocabulary; Exp.lang = Expressive vocabulary, RJA = Responding to joint attention; IJA = Initiating joint attention; PLS = Parental supportive behaviors. Age is measured by lab visit number.

**Table SI2.**

*Conditional effects of responding to joint attention on receptive vocabulary at varying levels of parental supportive behaviors (PSB)*

| *Variable*  PSB | *Effect* | *SE* | *t* | *p-value* | *LLCI* | *ULCI* |
| --- | --- | --- | --- | --- | --- | --- |
| -.78 | 49.40 | 6.54 | 7.55 | .000 | 36.51 | 62.29 |
| .03 | 40.32 | 4.59 | 8.79 | .000 | 31.28 | 49.37 |
| .83 | 31.27 | 6.96 | 4.50 | .000 | 17.56 | 44.98 |
